# Beta-Hydroxysteroid Dehydrogenase Genes in Orange-Spotted Grouper (*Epinephelus coioides*): Genome-Wide Identification and Expression Analysis During Sex Reversal

**DOI:** 10.3389/fgene.2020.00161

**Published:** 2020-03-04

**Authors:** Ling Xiao, Yin Guo, Dengdong Wang, Mi Zhao, Xin Hou, Shuisheng Li, Haoran Lin, Yong Zhang

**Affiliations:** ^1^State Key Laboratory of Biocontrol, Guangdong Provincial Key Laboratory for Aquatic Economic Animals and Southern Marine Science and Engineering Guangdong Laboratory (Zhuhai), School of Life Sciences, Sun Yat-sen University, Guangzhou, China; ^2^Laboratory for Marine Fisheries Science and Food Production Processes, Qingdao National Laboratory for Marine Science and Technology, Qingdao, China; ^3^Southern Marine Science and Engineering Guangdong Laboratory (Zhanjiang), Fisheries College, Guangdong Ocean University, Zhanjiang, China; ^4^Marine Fisheries Development Center of Guangdong Province, Huizhou, China

**Keywords:** β-HSD, orange-spotted grouper (*Epinephelus coioides*), genome-wide analysis, gene expression, sex reversal

## Abstract

Beta-hydroxysteroid dehydrogenases (β-HSDs) are a group of steroidogenic enzymes that are involved in steroid biosynthesis and metabolism, and play a crucial role in mammalian physiology and development, including sex determination and differentiation. In the present study, a genome-wide analysis identified the numbers of β*-hsd* genes in orange-spotted grouper (*Epinephelus coioides*) (19), human (*Homo sapiens*) (22), mouse (*Mus musculus*) (24), chicken (*Gallus gallus*) (16), xenopus (*Xenopus tropicalis*) (24), coelacanth (*Latimeria chalumnae*) (17), spotted gar (*Lepisosteus oculatus*) (14), zebrafish (*Danio rerio*) (19), fugu (*Takifugu rubripes*) (19), tilapia (*Oreochromis niloticus*) (19), medaka (*Oryzias latipes*) (19), stickleback (*Gasterosteus aculeatus*) (17) and common carp (*Cyprinus carpio*) (27) samples. A comparative analysis revealed that the number of β*-hsd* genes in teleost fish was no greater than in tetrapods due to gene loss followed by a teleost-specific whole-genome duplication event. Based on transcriptome data from grouper brain and gonad samples during sex reversal, six β*-hsd* genes had relatively high expression levels in the brain, indicating that these genes may be required for neurogenesis or the maintenance of specific biological processes in the brain. In the gonad, two and eight β*-hsd* genes were up- and downregulated, respectively, indicating their important roles in sex reversal. Our results demonstrated that β*-hsd* genes may be involved in the sex reversal of grouper by regulating the synthesis and metabolism of sex steroid hormones.

## Introduction

In almost all vertebrates, sexual reproduction requires both females and males to maintain survival and variation. Therefore, sex determination and differentiation are important processes for the continuation of species (Kobayashi et al., 2013). The orange-spotted grouper (*Epinephelus coioides*) is a protogynous hermaphrodite species. The gonads of most groupers develop into ovaries during the first sex differentiation; however, the ovaries of some individuals then change to testis after female sexual maturation is complete in a process known as sex reversal (Liu and de Mitcheson, 2009). Gonadal changes in sex-changing fish are accompanied by changes in plasma levels of sex steroids (Godwin, 2010), and the balance between estrogen and androgen controls the direction of gonadal development during sex reversal (Liu et al., 2017).

All classes of steroid hormones are synthesized from the common precursor cholesterol. The synthesis of sex steroid hormones is carried out under the catalysis of a series of enzymes, and the genes encoding these enzymes include *hsd3b1*, *hsd11b2*, *hsd17b1*, *hsd17b3*, and *hsd20b2* of the beta-hydroxysteroid dehydrogenases (β-HSDs) superfamily, and *cyp11a1*, *cyp11b*, *cyp17a1*, *cyp19a1a*, and *cyp21a1* of the cytochrome P450 (cP450) superfamily (Tokarz et al., 2015). Hsd3b1 is responsible for the oxidation and isomerization of Δ5-3β-hydroxysteroid precursors in order to form Δ4-3-ketosteroid (Nakamoto et al., 2012). Hsd11b2 catalyzes the conversion of cortisol to cortisone (Fuller et al., 2012), and also plays a crucial role in the conversion of 11β-OH-androstenedione to 11-ketotestosterone (11-KT) (Tokarz et al., 2015), which affects the production of the most important androgen in teleost fish. Furthermore Hsd17b1 converts inactive estrone to active estradiol (Mindnich et al., 2004), Hsd17b3 is an essential enzyme for the synthesis of 11-KT (Mindnich et al., 2005) and Hsd20b2 converts 17alpha-hydroxyprogesterone to 17alpha,20beta-DP, which has been identified as the maturation-inducing hormone of several teleosts (Senthilkumaran et al., 2002).

HSDs catalyze the conversion of a hydroxy group to a keto group or vice versa on a steroid ring in a specific position and orientation (Yang et al., 2005). Different types of β-HSDs are found in various tissues including brain and gonad (He et al., 2018), and most belong to the short chain dehydrogenase/reductase (SDR) family, except for HSD17B5, which belongs to the structural family of aldo-keto reductases (AKRs) (Day et al., 2008). To the best of our knowledge, systematic identification of the SDR and HSD gene families has only been performed in plants (Moummou et al., 2012) and bacteria (Kisiela et al., 2012), respectively. In addition, functional studies of β*-hsd* genes have mainly utilized human, mouse and zebrafish model organisms, while little is known about their expression and function in other species.

Orange-spotted grouper is an important marine fish cultured in China and Southeast Asian countries. Recently, sequencing and assembly of the grouper genome, and RNA-sequencing of the brain and gonad of grouper during sex reversal were completed (unpublished data), making it a good model for genome-wide identification and gonadal expression profiling of β*-hsd* genes.

Here we report the genome-wide identification of β*-hsd* genes in grouper, human (*Homo sapiens*), mouse (*Mus musculus*), chicken (*Gallus gallus*), xenopus (*Xenopus tropicalis*), coelacanth (*Latimeria chalumnae*), spotted gar (*Lepisosteus oculatus*), zebrafish (*Danio rerio*), fugu (*Takifugu rubripes*), tilapia (*Oreochromis niloticus*), medaka (*Oryzias latipes*), stickleback (*Gasterosteus aculeatus*), and common carp (*Cyprinus carpio*). Furthermore, RNA-seq data was used to analyze the expression of β*-hsd* genes during the sex reversal of grouper. Our study may provide a greater understanding of the evolution and possible functions of β*-hsd* genes in teleosts.

## Materials and Methods

### Sample Preparation and Gonadal Classification

All animal experiments were conducted in accordance with the guidelines and approval of the Animal Research and Ethics Committees of Sun Yat-sen University. Grouper were cultivated in the Marine Fisheries Development Center of Guangdong Province. In April 2015, 100 groupers weighing approximately 5 kg were sacrificed for tissue sampling after being anesthetized by MS222. For each fish, the gonad and brain were taken and stored at −80°C for RNA extraction, and a piece of gonadal tissue was fixed in Bouin’s fluid for histological examination.

Histological examination was performed as described previously (Xiao et al., 2018). Each gonad was assigned to one of five periods based on the gonadal morphology previously reported (Liu and de Mitcheson, 2009): ovarian-phase-2 (Ov2), ovarian-phase-3 (Ov3), ovarian-phase-4 (Ov4), bisexual-phase (Bi) and testis (Te). Ov2 gonad was predominated by primary-growth stage oocytes, Ov3 gonad was accompanied by the development of cortical-alveolus stage oocytes, and the appearance of vitellogenic stage oocytes indicated that the ovary had matured and belonged to Ov4. During the sex reversal of grouper, spermatogenic cysts and primary-growth stage oocytes are present in the bisexual-phase gonad simultaneously. The oocyte completely disappears and the gonad is filled with male germ cells, indicating that the sex reversal has been completed and the gonad has changed to testis.

### Genome-Wide Identification of β*-hsd* Genes

The genome sequences and predicted protein sequences of human^1^, mouse^2^, chicken^3^, xenopus^4^, coelacanth^5^, spotted gar^6^, zebrafish^7^, fugu^8^, tilapia^9^, medaka^10^, and stickleback^11^ were downloaded from Ensembl, and the sequences of the common carp were downloaded from NCBI^12^. The genomic sequence of grouper has been recently sequenced, with 1.03 GB of genomic data assembled and 23043 encoded genes predicted, of which 23,013 genes could be annotated (PRJEB28248 in the NCBI Sequence Read Archive site).

To identify candidate β*-hsd* genes in these thirteen species, we collected the gene and protein names of the β*-hsd* gene family from the literature (Payne et al., 1997; Dai et al., 2003; Mindnich et al., 2004; Seckl, 2004; Hoffmann and Maser, 2007; Meier et al., 2009; Mindnich and Adamski, 2009; Sreenivasulu and Senthilkumaran, 2009; Rotinen et al., 2010; Kisiela et al., 2012; Saloniemi et al., 2012; Tokarz et al., 2012; Lin et al., 2013; He et al., 2018). Secondly, the amino acid sequences of human and zebrafish β*-hsd* genes were collected from Ensembl and GenBank by searching for their gene and protein names. Next, specific hidden Markov models (HMMs) of human and zebrafish β-HSDs were developed by hmmbuild command in HMMER (HMMER3.1B2^13^) and then used as the query sequences to search the protein databases of all the studied species with hmmsearch command in HMMER (*E*-value = 0.01). Because all the β*-hsd* genes belong to the SDR family, except for *hsd17b5*, which belongs to the AKR superfamily, there was concern that the HMMs-based search would miss *hsd17b5*. Therefore, the amino acid sequence of human HSD17B5 was used as query sequence to search the protein databases of other species by local BLASTP with a cut off *E*-value of 1e-5 (BLAST 2.2.26 release from NCBI by anonymous FTP^14^) (Altschul et al., 1997). All the suspected sequences were aligned with the NCBI non-redundant protein sequence database by online BLASTP program to obtain reliable sequences of β*-hsd* genes.

To explore the structural diversity of the β-HSD family, the 19 grouper β-HSD protein sequences were submitted to the online CD-Search in CDD program^15^ (Marchler-Bauer et al., 2017) to search for functional domains (*E*-value = 0.01). In addition, the online tool GSDS 2.0^16^ (Hu et al., 2015) was used to display the gene structure and functional domains of grouper β*-hsd* genes.

### Phylogenetic Analysis

Multiple alignment of the amino acid sequences of all β-HSDs from six species including grouper, human, mouse, zebrafish, tilapia, and stickleback was performed using ClustalX-2.1 program (Jeanmougin et al., 1998). The maximum likelihood tree and neighbor-joining phylogenetic trees were constructed using the MEGA7 program (Kumar et al., 2016).

### Transcriptome-Based Analysis of Expression Profiling of the β*-hsd* Genes

Brain and gonad tissue from two fish in each stage were selected for transcriptome sequencing. RNA extraction and detection, library construction, sequencing, gene expression calculation and differential gene expression analyses were performed as previously described (Xiao et al., 2018). These RNA-seq data (PRJNA564153 in the NCBI Sequence Read Archive site) were used to analyze the β*-hsd* gene expression profiles during sex reversal. The expression of β*-hsd* genes was shown using R packages (i386 3.4.0). The RPKM values were transformed into Z scores before drawing heatmap. Z scores were plotted according to *Z* = (x - μ)/σ, where x is the log2 transformed gene expression measurement and μ and σ are the mean and standard deviations of expression of the gene.

### Gene Expression Profiling by Quantitative Real-Time PCR (qRT-PCR)

To examine the reliability of RNA-seq results, 10 β*-hsd* genes were selected for validation using qRT-PCR. Because there were only four bisexual-phase fish among the 100 sacrificed individuals, four fish were used for qRT-PCR in each period. Total RNA was extracted and reverse-transcribed using TRIzol^TM^ reagent (Invitrogen, United States) and a Transcriptor First Strand cDNA Synthesis Kit (Roche, United States), respectively. Specific primers (Supplementary Table S3) were designed by Primer Premier 6 software. β*-actin* was chosen to be the housekeeping gene for its stable expression in brain and gonad tissue during sex reversal. The qRT-PCR reactions based on SYBR (LightCycler 480 SYBR Green I Master, Roche, United States) were performed with a LightCycler 480 system (Roche, United States). Relative expression levels were calculated using the 2^–△^^△^^*Ct*^ method (Livak and Schmittgen, 2001). Analysis and visualization of quantitative results was performed by GraphPad Prism 6.0, and one-way ANOVA statistical analysis was used in the analysis.

## Results

### Identification of β*-hsd* Genes

**FIGURE 1:**
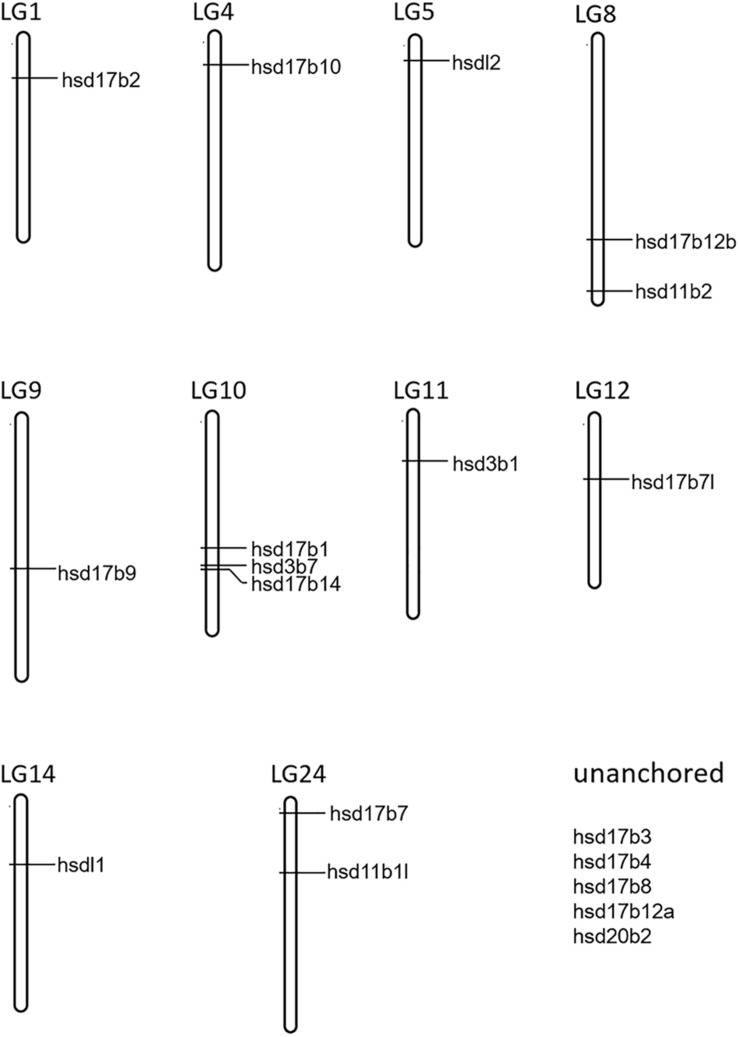
Chromosome localization of 19 β*-hsd* genes from orange-spotted grouper.

A total of 22 and 19 β*-hsd* genes were collected from the human and zebrafish genome, respectively, which were used to construct HMM models. A set of 19 putative members of the β*-hsd* genes have been identified from the orange-spotted grouper genome (Table 1). Among the 19 β*-hsd* genes, a total of 14 were distributed across 10 linkage groups (LGs) in the grouper genome (Figure 1). In addition, we also identified 24, 16, 24, 17, 14, 19, 19, 19, 17, and 27 β*-hsd* genes in mouse, chicken, xenopus, coelacanth, spotted gar, fugu, tilapia, medaka, stickleback, and common carp, respectively (Table 1). The gene and protein IDs of the β*-hsd* genes of all species are listed in the attached table (Supplementary Table S1).

**TABLE 1:** Number variation of β*-hsd* genes in the orange-spotted grouper and the other surveyed animals.

	Human	Mouse	Chicken	Xenopus	Coelacanth	Spotted gar	Zebrafish	Fugu	Tilapia	Medaka	Stickleback	Common carp	Grouper	Total
*hsd3b1*	1	1	1	1	1	1	1	1	1	1	1	1	1	13
*hsd3b2*	1	1	–	–	–	–	1	–	–	–	–	–	–	3
*hsd3b3*	–	1	–	–	–	–	–	–	–	–	–	–	–	1
*hsd3b4*	–	1	–	–	–	–	–	–	–	–	–	–	–	1
*hsd3b5*	–	1	–	–	–	–	–	–	–	–	–	–	–	1
*hsd3b6*	–	1	–	–	–	–	–	–	–	–	–	–	–	1
*hsd3b7*	1	1	1	2	1	1	1	1	1	1	1	2	1	15
*hsd11b1*	2	1	3	3	1	1	1	1	2	2	1	2	1	21
*hsd11b2*	1	1	1	1	1	1	1	1	1	1	1	3	1	15
*hsd17b1*	1	1	1	1	–	1	1	1	1	1	1	1	1	12
*hsd17b2*	1	1	1	1	1	1	1	1	–	–	–	2	1	11
*hsd17b3*	1	1	1	1	1	1	1	1	1	1	1	2	1	14
*hsd17b4*	1	1	1	1	1	1	1	1	1	1	1	1	1	13
*hsd17b5*	1	–	–	–	–	–	–	–	–	–	–	–	–	1
*hsd17b6*	1	1	–	3	1	–	–	–	–	–	–	–	–	6
*hsd17b7*	1	1	1	1	1	1	1	2	2	2	1	1	2	17
*hsd17b8*	1	1	–	1	1		1	1	1	1	1	1	1	11
*hsd17b9*	1	1	–	–	–	1	1	1	1	1	1	–	1	9
*hsd17b10*	1	1	1	1	1	–	1	1	1	1	1	–	1	11
*hsd17b11*	1	1	1	1	1	–	–	–	–	–	–	–	–	5
*hsd17b12*	1	1	1	2	2	1	2	2	2	2	2	4	2	24
*hsd17b13*	1	1	–	1	–	–	–	–	–	–	–	–	–	3
*hsd17b14*	1	1	–	1	1	–	1	1	1	1	1	1	1	11
*hsd20b2*	–	–	–	–	–	1	1	1	1	1	1	2	1	9
*hsdl1*	1	1	1	1	1	1	1	1	1	1	1	2	1	14
*hsdl2*	1	1	1	1	1	1	1	1	1	1	1	2	1	14
total	22	24	16	24	17	14	19	19	19	19	17	27	19	256
														

### Gene Structure and Conserved Domains

**FIGURE 2:**
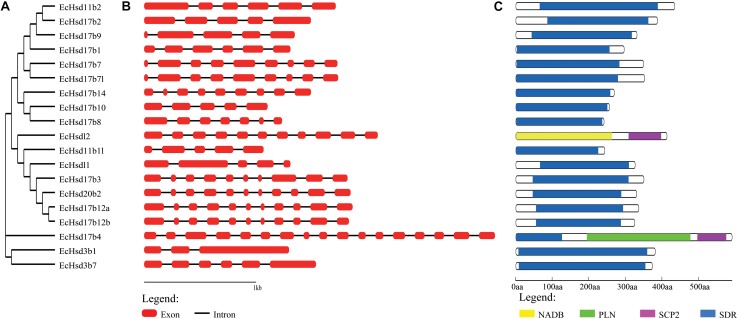
Phylogenetic analysis, gene structure and conserved domains of β*-hsd* genes in orange-spotted grouper. **(A)** The phylogenetic tree of all β-Hsd in grouper was constructed using Neighbor-Joining method. **(B)** The exon/intron organization of β*-hsd* genes of grouper. The red boxes represent exons and black lines indicate introns. **(C)** The conserved protein domains in the β-Hsd were identified using CD-Search program. Each domain is indicated with a specific color. Yellow: NADB_Rossmann superfamily, green: PLN02864 superfamily, purple: SCP2 superfamily, blue: SDR superfamily.

The exon-intron structure of the grouper β*-hsd* genes was further characterized, and showed that the number of introns in each β*-hsd* gene varied from 2–16 (Figure 2B). *Hsd3b1* was the gene with the lowest number of introns, containing only two, while *hsd17b4* had the greatest amount at 16. In addition, a phylogenetic tree was constructed using the grouper β-HSD protein sequences to determine whether the exon-intron structure is consistent with the phylogenetic tree (Figure 2A). As expected, several genes with similar exon/intron structures were clustered together on the phylogenetic tree, such as *hsd17b7*/*hsd17b7l*, *hsd20b2*/*hsd17b12a*/*hsd17b12b*. However, due to large number of exon/intron structure types of the grouper β*-hsd* genes, only a few genes shared similar structures.

The functional domains of grouper β-HSD were predicted by the CD-Search (Marchler-Bauer et al., 2017) based on their protein sequences (Figure 2C). As shown in Figure 2C, 18 of the 19 grouper β-HSDs possessed an SDR superfamily domain, except Hsdl2, indicating a high level of conservation of β*-hsd* genes belonging to the SDR superfamily. In addition to the SDR superfamily domain, Hsd17b4 also contained two other domains: PLN02864 superfamily domain and SCP2 superfamily domain. Notably, Hsdl2 was a member of the SDR superfamily, but no SDR superfamily domain existed in grouper Hsdl2, according to the CDD prediction. Therefore, we also predicted the functional domain of Hsdl2 protein sequences of other species by CD-Search (Supplementary Figure S1). As shown in Supplementary Figure S1, six Hsdl2 protein sequences contained the SDR superfamily domain and did not contain the NADB_Rossmann superfamily domain, while the other eight Hsdl2 protein sequences, including grouper Hsdl2, were the opposite.

### Phylogenetic Analysis

**FIGURE 3:**
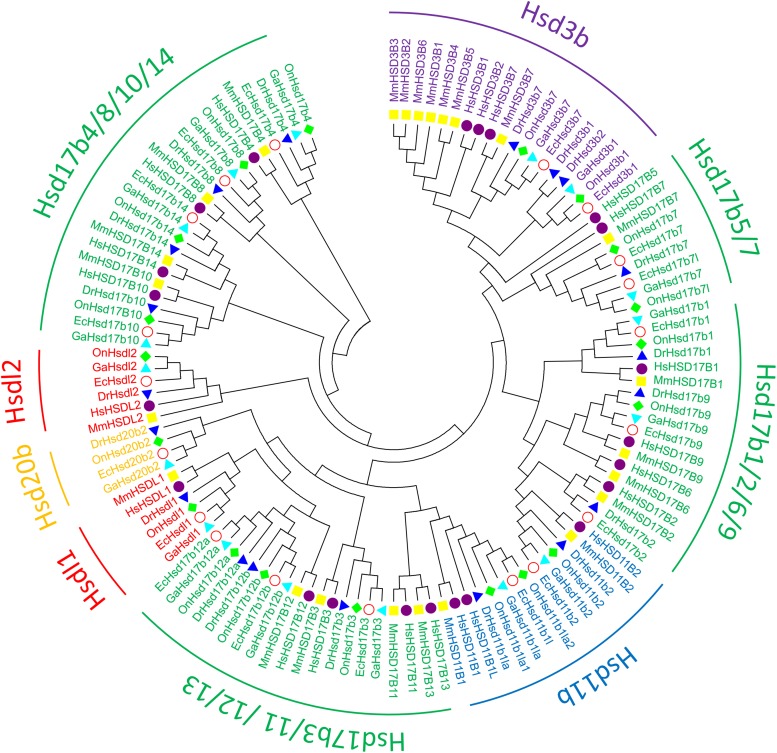
Phylogenetic relationships of β-Hsd proteins from grouper and other five species. The phylogenetic tree was constructed using MEGA7 by Maximum Likelihood method with the amino acid sequences of β-Hsd from grouper (*Epinephelus coioides*, Ec), (*Homo sapiens*, Hs), mouse (*Mus musculus*, Mm), zebrafish (*Danio rerio*, Dr), tilapia (*Oreochromis niloticus*, On), and stickleback (*Gasterosteus aculeatus*, Ga).

To clarify the evolutionary relationships among β*-hsd* genes, a phylogenetic tree was constructed with amino acid sequences of 96 β-HSDs collected from grouper, human, zebrafish, stickleback, and tilapia. Both maximum likelihood (ML, Figure 3) and Neighboring-Joining method (NJ, Supplementary Figure S2) strategies were devoted to construct phylogenetic trees based on the alignment of their amino acid sequences. The trees produced by these two methods differed only in a small number of branches, indicating that the evolutionary tree is credible.

### Expression of β*-hsd* Genes During Sex Reversal of Grouper

**FIGURE 4:**
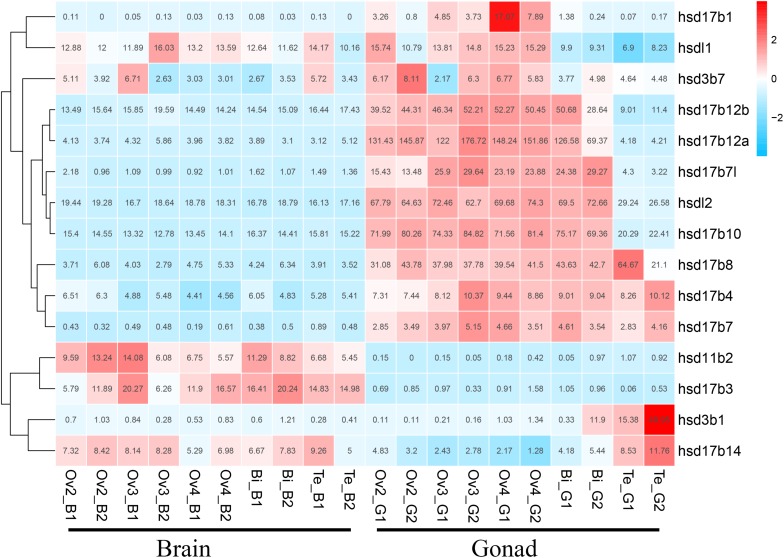
Clustering of the expression profiles of 19 grouper β*-hsd* genes during sex reversal. Genes were clustered according to phylogenetic relationships in expression profiles. The RPKM values were transformed into Z scores. Z scores were plotted according to *Z* = (x - μ)/σ, where x is the log 2 transformed gene expression measurement and μ and σ are the mean and standard deviations of expression of the gene. Red and blue colors indicate high and low relative expression levels after homogenization, respectively. The RPKM value for each gene and each stage was provided in the figure.

We used transcriptome data of grouper brains and gonads at five developmental stages, namely Ov2(ovarian-phase-2), Ov3(ovarian-phase-3), Ov4(ovarian-phase-4), Bi(bisexual-phase) and Te(testis), to profile the expression of β*-hsd* genes during sex reversal of grouper. By transcriptomic analysis, 18 of the 19 β*-hsd* genes were detected in the brains and gonads during sex reversal, except for *hsd17b2* (Supplementary Table S2). According to the criteria that a gene is considered to be expressed if it exhibits an expression level with RPKM value ≥2, we found that except for *hsd11b1l*, *hsd17b9*, and *hsd20b2*, the other 15 β*-hsd* genes were expressed in the brains or gonads in at least one development stage (Figure 4 and Supplementary Table S2). Among these expressed β*-hsd* genes, eleven members (*hsd3b1*, *hsd17b1*, *hsd17b4*, *hsd17b7l*, *hsd17b8*, *hsd17b10*, *hsdl17b12a*, *hsd17b12b*, *hsd17b14*, *hsdl1*, and *hsdl2*) and six members (*hsd11b2*, *hsd17b3*, *hsd17b10*, *hsd17b12b*, *hsdl1*, and *hsdl2*) presented high expression (RPKM value ≥10) in gonads and brain in at least one developmental stage, respectively. As shown in Figure 4, the expression levels of eight β*-hsd* genes (*hsd3b7*, *hsd17b1*, *hsd17b7l*, *hsd17b10*, *hsd17b12a*, *hsd17b12b*, *hsdl1*, and *hsdl2*) were reduced, while two β*-hsd* genes (*hsd3b1* and *hsd17b14*) were up-regulated in the gonad during sex reversal.

**FIGURE 5:**
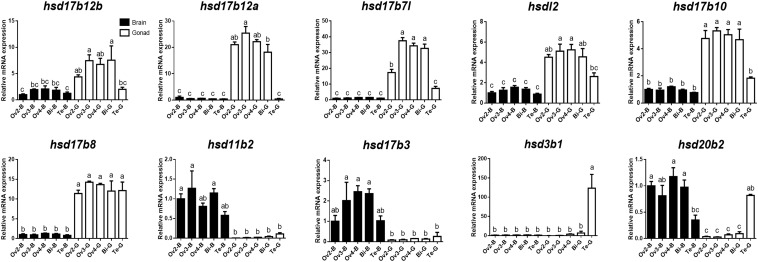
Quantitative RT-PCR examination of 10 β*-hsd* genes expressions in the brain and gonad during sex reversal of grouper. Values represent the relative mRNA expression in relation to internal control (β*-actin* gene). Data were expressed as the mean ± SEM for four replicates. In each panel, different lowercase letters indicate significant differences (*p* < 0.05).

To further verify the expression levels of β*-hsd* genes during sex reversal of grouper, qRT-PCR validation experiments were conducted using gene-specific primers (Supplementary Table S3). The four genes involved in the synthesis of sex steroid hormones (*hsd3b1*, *hsd11b2*, *hsd17b3*, and *hsd20b2*) and eight genes with RPKM value ≥20 (a total of ten genes after de-duplication) were selected for qRT-PCR validation. Expression patterns during sex reversal of these 10 genes were shown to be consistent with transcriptome data (Figures 4, 5 and Supplementary Table S2).

## Discussion

HSDs are a group of steroidogenic enzymes that are involved in steroid biosynthesis and metabolism (Ye et al., 2014). To the best of our knowledge, HSD gene repertoires have previously been described in bacteria (Kisiela et al., 2012), plants (Moummou et al., 2012), zebrafish (Mindnich et al., 2004; Mindnich and Adamski, 2009) and several mammals (Payne et al., 1997; Baker, 2001; Belyaeva and Kedishvili, 2006; Rotinen et al., 2010), but no studies have summarized all the β*-hsd* genes. Little is known about the corresponding grouper genes; however, the availability of the complete grouper genome sequence has made it possible to identify all the β*-hsd* genes in this species for the first time. In the present study, we systematically idntified 19 putative β*-hsd* genes in the *E. coioides* genome, and subsequently characterized the genes in terms of position in the genome, exon-intron structure and conserved domains. In addition, we also isolated β*-hsd* genes in samples of human, mouse, chicken, xenopus, coelacanth, spotted gar, zebrafish, fugu, tilapia, medaka, stickleback, and common carp. Finally, an analysis of grouper β*-hsd* gene expression levels during sex reversal indicated that these genes may play important roles in this significant physiological process. Our study represented the first comprehensive investigation of the fish β*-hsd* gene family, and the resulting data will undoubtedly be useful for future analyses, including further research into the role of β*-hsd* genes and sex steroids in sex reversal of grouper.

Comparative analysis revealed several evolutionary phenomena of the β*-hsd* genes during the separation of fish species from other animals. First, unlike some other gene families which have undergone an expansion in the teleost fish, the number of β*-hsd* genes in teleost fish was no greater than in tetrapods. Studies have reported after two major radiations of jawed vertebrate, teleosts shared another round of whole-genome duplication events (the 3R WGD) (Kasahara et al., 2007), and common carp underwent an additional whole-genome duplication event (the 4R WGD) (Xu et al., 2014). For the nuclear receptor (NR) superfamily, 48, 75, and 137 members have been reported in human, tilapia and common carp, respectively, suggesting that NRs may have expanded along with multiple duplication events (Cheng et al., 2015). The *sox* gene family has also undergone a continuous expansion in the teleost fish following their whole genome duplication (Wei et al., 2016). However, for the β*-hsd* genes, expansion after the 3R WGD and the 4R WGD was not as obvious as in the NR gene family and *sox* gene family. For example, *hsd3b1* and *hsd17b4* had only one copy in all species, and *hsd17b3*, *hsdl1*, and *hsdl2* had two copies in common carp which had undergone the 4R WGD, and only one copy in other species. Such a phenomenon is considered to be a result of gene loss followed by the WGD. The duplication-degeneration-complementation (DDC) model proposed by Lynch and Conery (2000) on the fate of duplicated genes suggested that the common fate of a duplicated gene was lost from the genome owing to non-functionalization unless one of the duplicate genes acquired a new function, or the functions of the ancestral gene are sub-divided between the sister duplicate genes. Gene loss was thought to be a mechanism to maintain a balance for duplicated genes post genome duplication events. Gene loss is evident in the β*-hsd* gene family, but not so obvious in the NR and *sox* gene families, which may be due to different selective pressures or different evolutionary rates among different gene catalogs. Secondly, several genes were identified only in a particular species. For instance, among all the species we studied, *Hsd3b3*, *Hsd3b4*, *Hsd3b5*, and *Hsd3b6* were specifically identified in the mouse genome. The six mouse *Hsd3b* genes (*MnHsd3b1*, *MnHsd3b2* and four mouse-specific *Hsd3b* genes) were located in a small region (98499071-98859794) of chromosome 3 and clustered together in the phylogenetic tree, seemingly suggesting that the four mouse-specific *Hsd3b* genes were more likely a product of mouse-specific gene duplication rather than gene loss in other species. In addition, *hsd20b2* was not identified in tetrapod genomes, apparently indicating that *hsd20b2* may be lost in the tetrapod genome but retained in the teleost genome following their divergence.

The orange-spotted grouper is a typical protogynous hermaphrodite species. Previous studies have shown that groupers in the natural environment generally develop into female individuals during the first sex differentiation (Liu and de Mitcheson, 2009). Then the sex reversal from female to male occur in a small part of mature/functional females, while the males retain the same sex throughout their life span (Bhandari et al., 2003). Sex steroids play a very important role in sex reversal of groupers. Low serum E2 levels and increased 11-KT levels are probably the events mediating protogynous sex change in grouper (Bhandari et al., 2003). In addition, exogenous androgens could artificially induce the sex reversal of groupers from female to male (Chen et al., 2011; Oh et al., 2013; Murata et al., 2014). However, little is known about the expression profiles of sex steroid synthesis and metabolism genes in the natural sex reversal process. HSDs participate in the synthesis and metabolism of sex steroid hormones by catalyzing the conversion of a hydroxy group to a keto group or vice versa on a steroid ring in a specific position and orientation (Yang et al., 2005). Studies on the expression profiles of *hsd* genes at different stages of grouper sex reversal may provide us a better understanding on the roles of *hsd* genes in sex reversal.

The transcriptome data indicated that six (*hsd11b2*, *hsd17b3*, *hsd17b10*, *hsd17b12b*, *hsdl1*, and *hsdl2*) and eleven (*hsd3b1*, *hsd17b1*, *hsd17b4*, *hsd17b7l*, *hsd17b8*, *hsd17b10*, *hsdl17b12a*, *hsd17b12b*, *hsd17b14*, *hsdl1*, and *hsdl2*) β*-HSD* genes had relatively high expression levels (RPKM >10) in the brain and gonad during sex reversal of grouper, respectively. HSDs are indispensable neuro-steroidogenic enzymes, and the neuro-steroids synthesized in neurons and glia can act on various receptors to regulate neuroexcitability while also playing important roles in brain development, neuroprotection and neurogenesis as well as cognition and memory (He et al., 2018). For example, human HSD17B10 is found in various brain regions and is essential for the maintenance of neurosteroid homeostasis (He et al., 2018); and Hsd11b2 is involved in the negative feedback regulation of cortisol post stress in the brain of zebrafish (Alderman and Vijayan, 2012). In grouper, transcriptome data has shown that the expression levels of six β*-hsd* genes highly expressed in the brain did not change significantly during sexual reversal, seemingly indicating that these genes may be required for neurogenesis or the maintenance of specific biological processes in the brain, but not for sex reversal events.

In the gonad, two (*hsd3b1* and *hsd17b14*) and eight (*hsd3b7*, *hsd17b1*, *hsd17b7l*, *hsd17b10*, *hsd17b12a*, *hsd17b12b*, *hsdl1*, and *hsdl2*) β*-HSD* genes were up- and downregulated during sex reversal, respectively. Hsd3b1 catalyzes the second step of steroidogenesis and is required for the synthesis of all steroids including 11-KT (Hsu et al., 2009). In human testis specimens, HSD17B14 protein has shown immunoreactivity in most of the seminiferous epithelium as well as in peritubular areas harboring Leydig cells (Sivik et al., 2012). Transfection of *HSD17B14* in human breast cancer cells significantly decreased the levels of estradiol (Jansson et al., 2006), and further studies have suggested a role for HSD17B14 in the local inactivation of steroid (Lukacik et al., 2007). Therefore, we hypothesized that the increased expression levels of *hsd3b1* during the sex reversal of grouper may promote the synthesis of 11-KT, while the increased expression level of *hsd17b14* may lead to the inactivation of estradiol in this study.

HSD17B1 is mainly expressed in the ovary (Mindnich and Adamski, 2009), and catalyzes lower estrogenic active estrone (E1) to highly active estradiol (E2) (Hakkarainen et al., 2015; Jarvensivu et al., 2015). HSD17B12 has been implicated in the conversion of estrone to estradiol as well as in the synthesis of arachidonic acid (AA), and plays important roles in ovarian function and female fertility (Kemilainen et al., 2016). Accordingly, we propose that the high expression levels of *hsd17b1*, *hsd17b12a* and *hsd17b 12b* in the ovary may be due to its involvement in the maintenance of the ovary, and the degeneration of ovary leads to the downregulation of these genes during the sex reversal grouper. The 3-beta-hydroxy-Delta(5)-C(27)-steroid oxidoreductase, which is encoded by *HSD3B7* gene, is a membrane-bound enzyme of the endoplasmic reticulum that catalyzes an early step in the synthesis of bile acids from cholesterol, and mutation of the *HSD3B7* gene causes neonatal cholestasis (Cheng et al., 2003). *Hsd17b7l* was identified only in grouper, fugu, tilapia and medaka in this study, and its function has not been studied yet. HSD17B10 catalyzes the oxidation of neuroactive steroids and degradation of isoleucine in the nervous system (Yang et al., 2014). *HSDL1* and *HSDL2* is highly expressed in human testis and ovary tissue (Huang et al., 2001; Dai et al., 2003). However, the function of Hsd3b7, Hsd17b7l, Hsd17b10, Hsdl1, and Hsdl2 in the gonad is not well understood. Therefore, further studies are required to reveal their functions in the gonad and their roles in the sex reversal of grouper.

## Conclusion

*The*β*-hsd* genes play important roles in the biosynthesis and metabolism of steroids. In the present study, a genome-wide analysis identified numbers of the β*-hsd* genes in the human (22), mouse (24), chicken (16), xenopus (24), coelacanth (17), spotted gar (14), zebrafish (19), fugu (19), tilapia (19), medaka (19), stickleback (17), grouper (19), and common carp (27) samples. A comparative analysis revealed that the number of β*-hsd* genes in teleost fish was no greater than in tetrapods due to gene loss followed by the teleost-specific whole-genome duplication event. Transcriptome-based expression profiling uncovered the expressions of the β*-hsd* genes during the sex reversal of grouper. The exact roles of these differentially expressed β*-hsd* genes during sex reversal need to be precisely characterized in the future.

## Ethics Statement

All animal experiments were conducted in accordance with the guidelines and approval of the Animal Research and Ethics Committees of Sun Yat-sen University.

## Author Contributions

LX performed the experiments and wrote the manuscript. YG and DW analyzed the data. MZ and XH contributed the reagents and materials. YZ, SL, and HL provided guidance on the whole manuscript. All authors reviewed and approved the final submission.

## Conflict of Interest

The authors declare that the research was conducted in the absence of any commercial or financial relationships that could be construed as a potential conflict of interest.

## Footnotes

1 http://asia.ensembl.org/Homo_sapiens/Info/Index

2 http://asia.ensembl.org/Mus_musculus/Info/Index

3 http://asia.ensembl.org/Gallus_gallus/Info/Index

4 http://asia.ensembl.org/Xenopus_tropicalis/Info/Index

5 http://asia.ensembl.org/Latimeria_chalumnae/Info/Index

6 http://asia.ensembl.org/Lepisosteus_oculatus/Info/Index

7 http://asia.ensembl.org/Danio_rerio/Info/Index

8 http://asia.ensembl.org/Takifugu_rubripes/Info/Index

9 http://asia.ensembl.org/Oreochromis_niloticus/Info/Index

10 http://asia.ensembl.org/Oryzias_latipes_hni/Info/Index

11 http://asia.ensembl.org/Gasterosteus_aculeatus/Info/Index

12 ftp://ftp.ncbi.nlm.nih.gov/genomes/Cyprinus_carpio/

13 http://www.hmmer.org/

14 ftp://ftp.ncbi.nlm.nih.gov/blast/executables/blast+/

15 https://www.ncbi.nlm.nih.gov/Structure/cdd/wrpsb.cgi

16 http://gsds.cbi.pku.edu.cn/
