# Supplementary material for: Beta-Hydroxysteroid Dehydrogenase Genes in Orange-Spotted Grouper (Epinephelus coioides): Genome-Wide Identification and Expression Analysis During Sex Reversal
Source: Front Genet. 2020 Mar 4;11:161. doi: 10.3389/fgene.2020.00161 (PMC7064643; doi:10.3389/fgene.2020.00161)
Supplement: Supplementary file 1 [file Data_Sheet_1.PDF]

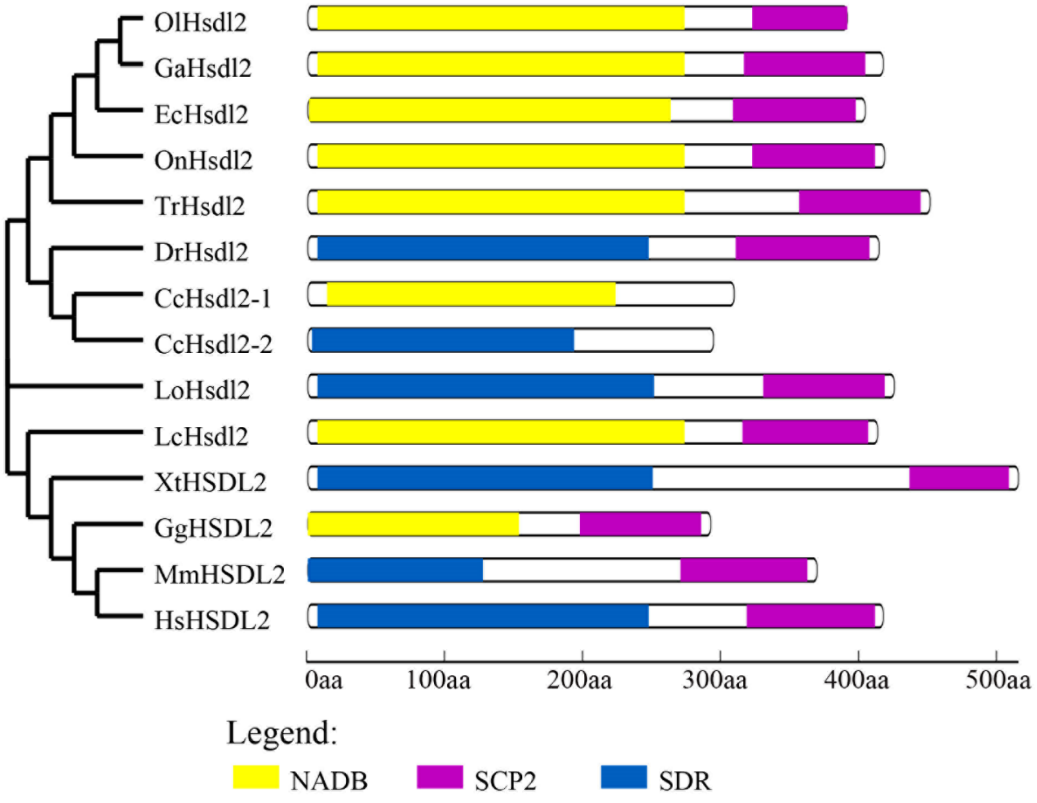

Figure S1. Phylogenetic analysis and conserved domains of Hsdl2 in grouper and other 12 species. The phylogenetic tree of all β-Hsd in grouper was constructed using Neighbor-Joining method, and the conserved protein domains were identified using CD-Search program. Each domain is indicated with a specific color. Yellow: NADB_Rossmann superfamily, purple: SCP2 superfamily, blue: SDR superfamily.

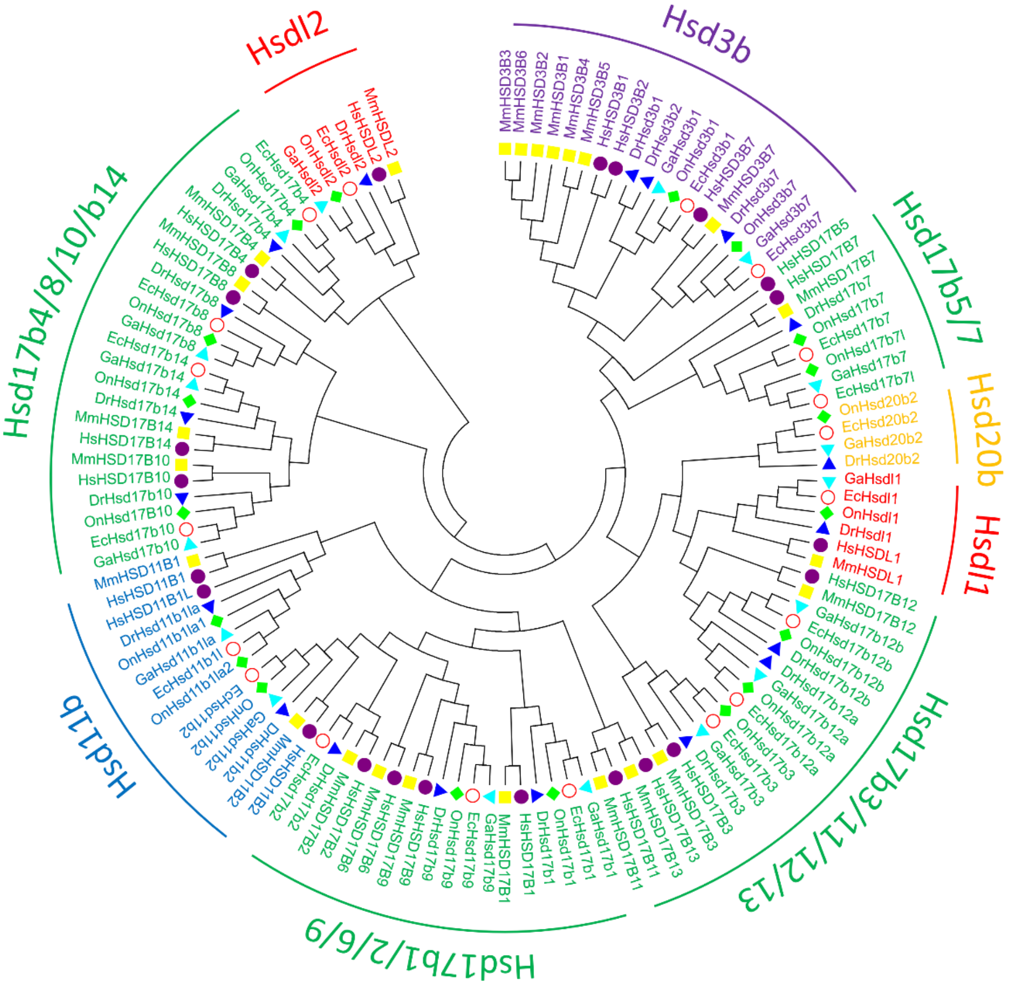

Figure S2. Phylogenetic relationships of β-Hsd proteins from grouper and other five species. The phylogenetic tree was constructed using MEGA7 by Neighbor-Joining method with the amino acid sequences of β-Hsd from grouper (*Epinephelus coioides*, Ec), (*Homo sapiens*, Hs), mouse (*Mus musculus*, Mm), zebrafish (*Danio rerio*, Dr), tilapia (*Oreochromis niloticus*, On) and stickleback (*Gasterosteus aculeatus*, Ga).

Table S1. The accession numbers of all the β-Hsd proteins identified in this study

Gene	Accession number	Gene	Accession number	Gene	Accession number
HsHSD3B1	ENSP00000432268	Lchsd3b1	ENSLACP00000017059	Onhsdl1	ENSONIP00000023066
HsHSD3B2	ENSP00000445122	Lchsd3b7	ENSLACP00000000808	Onhsdl2	ENSONIP00000015785
HsHSD3B7	ENSP00000297679	Lchsd11b1	ENSLACP00000021188	Olhsd3b1	ENSORLP00000010780
HsHSD11B1	ENSP00000355995	Lchsd11b2	ENSLACP00000005983	Olhsd3b7	ENSORLP00000002721
HsHSD11B1L	ENSP00000480443	Lchsd17b2	ENSLACP00000005652	Olhsd11b1la1	ENSORLP00000005684
HsHSD11B2	ENSP00000316786	Lchsd17b3	ENSLACP00000023218	Olhsd11b1la2	ENSORLP00000005734
HsHSD17B1	ENSP00000225929	Lchsd17b4	ENSLACP00000003889	Olhsd11b2	ENSORLP00000000394
HsHSD17B2	ENSP00000199936	Lchsd17b6	ENSLACP00000021358	Olhsd17b1	ENSORLP00000005206
HsHSD17B3	ENSP00000364412	Lchsd17b7	ENSLACP00000022779	Olhsd17b3	ENSORLP00000005353
HsHSD17B4	ENSP00000420914	Lchsd17b8	ENSLACP00000014448	Olhsd17b4	ENSORLP00000004428
HsHSD17B5	ENSP00000369927	Lchsd17b10	ENSLACP00000017524	Olhsd17b7	ENSORLP00000018891
HsHSD17B6	ENSP00000452273	Lchsd17b11	ENSLACP00000000458	Olhsd17b7l	ENSORLP00000013563
HsHSD17B7	ENSP00000254521	Lchsd17b12	ENSLACP00000019314	Olhsd17b8	ENSORLP00000008217
HsHSD17B8	ENSP00000363794	Lchsd17b12l	ENSLACP00000003405	Olhsd17b9	ENSORLP00000007912
HsHSD17B9	ENSP00000257895	Lchsd17b14	ENSLACP00000000696	Olhsd17b10	ENSORLP00000020907
HsHSD17B10	ENSP00000168216	Lchsdl1	ENSLACP00000010832	Olhsd17b12a	ENSORLP00000001758
HsHSD17B11	ENSP00000351035	Lchsdl2	ENSLACP00000012557	Olhsd17b12b	ENSORLP00000001233
HsHSD17B12	ENSP00000278353	Lohsd3b1	ENSLOCP00000013462	Olhsd17b14	ENSORLP00000001963
HsHSD17B13	ENSP00000333300	Lohsd3b7	ENSLOCP00000004068	Olhsd17b20b2	ENSORLP00000009883
HsHSD17B14	ENSP00000263278	Lohsd11b1lb	ENSLOCP00000006567	Olhsdl1	ENSORLP00000022038
HsHSDL1	ENSP00000219439	Lohsd11b2	ENSLOCP00000009941	Olhsdl2	ENSORLP00000024468
HsHSDL2	ENSP00000381785	Lohsd17b1	ENSLOCP00000015356	Gahsd3b1	ENSGACP00000001845
MmHsd3b1	ENSMUSP00000102630	Lohsd17b2	ENSLOCP00000002527	Gahsd3b7	ENSGACP00000018081
MmHsd3b2	ENSMUSP00000102636	Lohsd17b3	ENSLOCP00000011257	Gahsd11b1la	ENSGACP00000017030

MmHsd3b3	ENSMUSP00000102633	Lohsd17b4	ENSLOCP00000010734	Gahsd11b2	ENSGACP00000023138
MmHsd3b4	ENSMUSP00000136588	Lohsd17b7	ENSLOCP00000008624	Gahsd17b1	ENSGACP00000011285
MmHsd3b5	ENSMUSP00000041442	Lohsd17b9	ENSLOCP00000005926	Gahsd17b3	ENSGACP00000009250
MmHsd3b6	ENSMUSP00000029463	Lohsd17b12a	ENSLOCP00000001376	Gahsd17b4	ENSGACP00000008294
MmHsd3b7	ENSMUSP00000036245	Lohsd20b2	ENSLOCP00000016638	Gahsd17b7	ENSGACP00000021299
MmHsd11b1	ENSMUSP00000016338	Lohsdl1	ENSLOCP00000021340	Gahsd17b8	ENSGACP00000002411
MmHsd11b2	ENSMUSP00000034363	Lohsdl2	ENSLOCP00000003430	Gahsd17b9	ENSGACP00000007747
MmHsd17b1	ENSMUSP00000019445	Drhsd3b1	ENSDARP00000061320	Gahsd17b10	ENSGACP00000013944
MmHsd17b2	ENSMUSP00000034304	Drhsd3b2	ENSDARP00000025516	Gahsd17b12a	ENSGACP00000009205
MmHsd17b3	ENSMUSP00000132011	Drhsd3b7	ENSDARP00000112433	Gahsd17b12b	ENSGACP00000022633
MmHsd17b4	ENSMUSP00000025385	Drhsd11b1la	ENSDARP00000061738	Gahsd17b14	ENSGACP00000019385
MmHsd17b6	ENSMUSP00000151661	Drhsd11b2	ENSDARP00000118501	Gahsd20b2	ENSGACP00000026961
MmHsd17b7	ENSMUSP00000027989	Drhsd17b1	ENSDARP00000026566	Gahsdl1	ENSGACP00000009038
MmHsd17b8	ENSMUSP00000038069	Drhsd17b2	ENSDARP00000066984	Gahsdl2	ENSGACP00000020369
MmHsd17b9	ENSMUSP00000026406	Drhsd17b3	ENSDARP00000124637	Cchsd3b1	XP_018925427.1
MmHsd17b10	ENSMUSP00000108236	Drhsd17b4	ENSDARP00000136011	Cchsd3b7-1	XP_018927542.1
MmHsd17b11	ENSMUSP00000113455	Drhsd17b7	ENSDARP00000098015	Cchsd3b7-2	XP_018934920.1
MmHsd17b12	ENSMUSP00000028619	Drhsd17b8	ENSDARP00000001459	Cchsd11b1l-1	XP_018921668.1
MmHsd17b13	ENSMUSP00000046772	Drhsd17b9	ENSDARP00000013032	Cchsd11b1l-2	XP_018963376.1
MmHsd17b14	ENSMUSP00000103381	Drhsd17b10	ENSDARP00000017597	Cchsd11b2	XP_018924670.1
MmHsdl1	ENSMUSP00000044371	Drhsd17b12a	ENSDARP00000014003	Cchsd11b2l-1	XP_018926965.1
MmHsdl2	ENSMUSP00000103152	Drhsd17b12b	ENSDARP00000089612	Cchsd11b2l-2	XP_018932885.1
GgHsd3b1	ENSGALP00000051482	Drhsd17b14	ENSDARP00000071579	Cchsd17b1	XP_018954756.1
GgHsd3b7	ENSGALP00000060724	Drhsd20b2	ENSDARP00000091542	Cchsd17b2l-1	XP_018970521.1
GgHsd11b1a	ENSGALP00000002086	Drhsdl1	ENSDARP00000061172	Cchsd17b2l-2	XP_018970527.1
GgHsd11b1b	ENSGALP00000042266	Drhsdl2	ENSDARP00000129592	Cchsd17b3-1	XP_018964213.1

GgHsd11b1l	ENSGALP00000020944	Trhsd3b1	ENSTRUP00000040858	Cchsd17b3-2	XP_018971363.1
GgHsd11b2	ENSGALP00000042090	Trhsd3b7	ENSTRUP00000021824	Cchsd17b4l	XP_018970268.1
GgHsd17b1	ENSGALP00000041465	Trhsd11b1la	ENSTRUP00000046072	Cchsd17b7l	XP_018960707.1
GgHsd17b2	ENSGALP00000008766	Trhsd11b2	ENSTRUP00000019012	Cchsd17b8	XP_018979734.1
GgHsd17b3	ENSGALP00000062109	Trhsd17b1	ENSTRUP00000038837	Cchsd17b12a-1	XP_018933276.1
GgHsd17b4	ENSGALP00000003418	Trhsd17b2	ENSTRUP00000006462	Cchsd17b12a-2	XP_018936083.1
GgHsd17b7	ENSGALP00000057452	Trhsd17b3	ENSTRUP00000037530	Cchsd17b12b-1	XP_018920176.1
GgHsd17b10	ENSGALP00000052520	Trhsd17b4	ENSTRUP00000042960	Cchsd17b12b-2	XP_018937767.1
GgHsd17b11	ENSGALP00000017865	Trhsd17b7	ENSTRUP00000046998	Cchsd17b14	XP_018937015.1
GgHsd17b12	ENSGALP00000054930	Trhsd17b7l	ENSTRUP00000028025	Cchsd20b2-1	XP_018960136.1
GgHsdl1	ENSGALP00000005167	Trhsd17b8	ENSTRUP00000012137	Cchsd20b2-2	XP_018953731.1
GgHsdl2	ENSGALP00000025208	Trhsd17b9	ENSTRUP00000047171	Cchsdl1-1	XP_018933014.1
XtHSD3B1	ENSXETP00000039206	Trhsd17b10	ENSTRUP00000045263	Cchsdl1-2	XP_018981981.1
XtHSD3B7	ENSXETP00000004063	Trhsd17b12a	ENSTRUP00000039333	Cchsdl2-1	XP_018935408.1
XtHSD3B7L	ENSXETP00000009753	Trhsd17b12b	ENSTRUP00000041560	Cchsdl2-2	XP_018935421.1
XtHSD11B1	ENSXETP00000019588	Trhsd17b14	ENSTRUP00000000556	Echsd3b1	Eco_gene_10002598
XtHSD11B1L	ENSXETP00000031363	Trhsd20b2	ENSTRUP00000041887	Echsd3b7	Eco_gene_10009505
XtHSD11B1L.2	ENSXETP00000026672	Trhsdl1	ENSTRUP00000007351	Echsd11b1l	Eco_gene_10004459
XtHSD11B2	ENSXETP00000010210	Trhsdl2	ENSTRUP00000016691	Echsd11b2	Eco_gene_10004416
XtHSD17B1	ENSXETP00000035101	Onhsd3b1	ENSONIP00000019759	Echsd17b1	Eco_gene_10002905
XtHSD17B2	ENSXETP00000007728	Onhsd3b7	ENSONIP00000012375	Echsd17b2	Eco_gene_10018650
XtHSD17B3	ENSXETP00000032475	Onhsd11b1la1	ENSONIP00000006124	Echsd17b3	Eco_gene_10010168
XtHSD17B4	ENSXETP00000038102	Onhsd11b1la2	ENSONIP00000006126	Echsd17b4	Eco_gene_10000727
XtHsd17B6P	ENSXETP00000009614	Onhsd11b2	ENSONIP00000004099	Echsd17b7	Eco_gene_10013242
XtHSD17B6	ENSXETP00000009607	Onhsd17b1	ENSONIP00000001509	Echsd17b7l	Eco_gene_10008628
XtHSD17B6L	ENSXETP00000009623	Onhsd17b3	ENSONIP00000019062	Echsd17b8	Eco_gene_10001457

XtHSD17B7	ENSXETP00000010307	Onhsd17b4	ENSONIP00000018247	Echsd17b9	Eco_gene_10020178
XtHSD17B8	ENSXETP00000043924	Onhsd17b7	ENSONIP00000003252	Echsd17b10	Eco_gene_10022314
XtHSD17B10	ENSXETP00000016841	Onhsd17b7l	ENSONIP00000023405	Echsd17b12a	Eco_gene_10019485
XtHSD17B11	ENSXETP00000038871	Onhsd17b8	ENSONIP00000025236	Echsd17b12b	Eco_gene_10011196
XtHSD17B12	ENSXETP00000048094	Onhsd17b9	ENSONIP00000008634	Echsd17b14	Eco_gene_10023514
XtHSD17B12L	ENSXETP00000032770	Onhsd17B10	ENSONIP00000020878	Echsd20b2	Eco_gene_10004251
XtHSD17B13	ENSXETP00000014066	Onhsd17b12a	ENSONIP00000019626	Echsdl1	Eco_gene_10018467
XtHSD17B14	ENSXETP00000009930	Onhsd17b12b	ENSONIP00000007419	Echsdl2	Eco_gene_10005510
XtHSDL1	ENSXETP00000054551	Onhsd17b14	ENSONIP00000012242		
XtHSDL2	ENSXETP00000063676	Onhsd20b2	ENSONIP00000005282		

Hs: *Homo sapiens*, Mm: *Mus musculus*, Gg: *Gallus gallus*, Xt: *Xenopus tropicalis*, Lc: *Latimeria chalumnae*, Lo: *Lepisosteus oculatus*, Dr: *Danio rerio*, Tr: *Takifugu rubripes*, On: *Oreochromis niloticus*, Ol: *Oryzias latipes*, Ga: *Gasterosteus aculeatus*, Cc: *Cyprinus carpio*, Ec: *Epinephelus coioides*.

Tabel S2 The RPKM value of grouper *β-hsd* genes

	Ov2_B1	Ov2_B2	Ov3_B1	Ov3_B2	Ov4_B1	Ov4_B2	Bi_B1	Bi_B2	Te_B1	Te_B2
hsd3b1	0.7	1.03	0.84	0.28	0.53	0.83	0.6	1.21	0.28	0.41
hsd3b7	5.11	3.92	6.71	2.63	3.03	3.01	2.67	3.53	5.72	3.43
hsd11b1l	0.05	0.16	0.09	0.14	0.09	0.04	0.16	0.06	0.17	0.22
hsd11b2	9.59	13.24	14.08	6.08	6.75	5.57	11.29	8.82	6.68	5.45
hsd17b1	0.11	0	0.05	0.13	0.03	0.13	0.08	0.03	0.13	0
hsd17b2	0.00	0.00	0.00	0.00	0.00	0.00	0.00	0.00	0.00	0.00
hsd17b3	5.79	11.89	20.27	6.26	11.9	16.57	16.41	20.24	14.83	14.98
hsd17b4	6.51	6.3	4.88	5.48	4.41	4.56	6.05	4.83	5.28	5.41
hsd17b7	0.43	0.32	0.49	0.48	0.19	0.61	0.38	0.5	0.89	0.48
hsd17b7l	2.18	0.96	1.09	0.99	0.92	1.01	1.62	1.07	1.49	1.36
hsd17b8	3.71	6.08	4.03	2.79	4.75	5.33	4.24	6.34	3.91	3.52
hsd17b9	0.82	0.40	0.40	0.00	0.46	0.46	1.07	0.18	0.56	0.11
hsd17b10	15.4	14.55	13.32	12.78	13.45	14.1	16.37	14.41	15.81	15.22
hsd17b12a	4.13	3.74	4.32	5.86	3.96	3.82	3.89	3.1	3.12	5.12
hsd17b12b	13.49	15.64	15.85	19.59	14.49	14.24	14.54	15.09	16.44	17.43
hsd17b14	7.32	8.42	8.14	8.28	5.29	6.98	6.67	7.83	9.26	5
hsd20b2	0.40	0.23	0.52	0.82	0.00	0.08	0.23	0.24	0.58	0.08
hsdl1	12.88	12	11.89	16.03	13.2	13.59	12.64	11.62	14.17	10.16
hsdl2	19.44	19.28	16.7	18.64	18.78	18.31	16.78	18.79	16.13	17.16

Table S2 Continued

	Ov2_G1	Ov2_G2	Ov3_G1	Ov3_G2	Ov4_G1	Ov4_G2	Bi_G1	Bi_G2	Te_G1	Te_G2
hsd3b1	0.11	0.11	0.21	0.16	1.03	1.34	0.33	11.9	15.38	49.95
hsd3b7	6.17	8.11	2.17	6.3	6.77	5.83	3.77	4.98	4.64	4.48
hsd11b1l	0.01	0.00	0.00	0.02	0.02	0.00	0.02	0.08	0.63	0.52
hsd11b2	0.15	0	0.15	0.05	0.18	0.42	0.05	0.97	1.07	0.92
hsd17b1	3.26	0.8	4.85	3.73	17.07	7.89	1.38	0.24	0.07	0.17
hsd17b2	0.00	0.00	0.00	0.00	0.00	0.00	0.00	0.00	0.00	0.00
hsd17b3	0.69	0.85	0.97	0.33	0.91	1.58	1.05	0.96	0.06	0.53
hsd17b4	7.31	7.44	8.12	10.37	9.44	8.86	9.01	9.04	8.26	10.12
hsd17b7	2.85	3.49	3.97	5.15	4.66	3.51	4.61	3.54	2.83	4.16
hsd17b7l	15.43	13.48	25.9	29.64	23.19	23.88	24.38	29.27	4.3	3.22
hsd17b8	31.08	43.78	37.98	37.78	39.54	41.5	43.63	42.7	64.67	21.1
hsd17b9	0.00	0.00	0.00	0.00	0.00	0.00	0.00	0.13	0.20	0.87
hsd17b10	71.99	80.26	74.33	84.82	71.56	81.4	75.17	69.36	20.29	22.41
hsd17b12a	131.43	145.87	122	176.72	148.24	151.86	126.58	69.37	4.18	4.21
hsd17b12b	39.52	44.31	46.34	52.21	52.27	50.45	50.68	28.64	9.01	11.4
hsd17b14	4.83	3.2	2.43	2.78	2.17	1.28	4.18	5.44	8.53	11.76
hsd20b2	0.00	0.32	0.00	0.19	0.00	0.00	0.00	0.00	0.42	0.56
hsdl1	15.74	10.79	13.81	14.8	15.23	15.29	9.9	9.31	6.9	8.23
hsdl2	67.79	64.63	72.46	62.7	69.68	74.3	69.5	72.66	29.24	26.58

Ov2: ovarian-phase-2, Ov3: ovarian-phase-3, Ov4: ovarian-phase-4, Bi: bisexual-phase, Te: testis, B: Brain, G: Gonad.

Table S3 The primers used in qRT-PCR.

Gene name	Forward	Reverse
hsd3b1	CGTCAGAGCTGCCGAAGGAG	TTTAGTTTTGACTGTTGCTG
hsd11b2	CGGCCTCATGTACTTCATCC	TGGTCCTCTATCTGCGGGTG
hsd17b3	CATACTTCTGGGCTGGTTAC	GACATTGCTGTTCCTGGTTA
hsd17b7l	CACTCAGCCTCGACAGATGG	GCGAAAGCGTCCCTTACAAA
hsd17b8	TGTGCTGCCAGGTTTCATAT	ACAGACAGCCGATTTATCCC
hsd17b10	CTGTTCTCCACTCCCCTCCT	TCCGTCCAGTCTGATGACCT
hsd17b12a	GTTGCGAGAACAGAAGAATGG	ATGAGCACGTTTACCGAAGG
hsd17b12b	AACAAAGGCATCATCATTCAGA	GCAGGGAGCAGAGCAGTAGTC
hsd20b2	AGGATAGGTATGGACGAAAG	GTCTGGTCATCTGAGGGACT
hsdl2	ACACGCCTTCAAACCACCAT	TCTTCAAGTCCAGGAACCAAA
β-actin	ACCATCGGCAATGAGAGGTT	ACATCTGCTGGAAGGTGGAC
